# Use and discontinuation of antipsychotic medication in 20 years following a first episode of schizophrenia: results from the OPUS trial

**DOI:** 10.1017/S0033291724002678

**Published:** 2024-11

**Authors:** Helene Gjervig Hansen, Helene Speyer, Anne Emilie Stürup, Carsten Hjorthøj, Dost Öngür, Merete Nordentoft, Nikolai Albert

**Affiliations:** 1 Copenhagen Research Centre for Mental Health – CORE, Mental Health Center Copenhagen, Mental Health Services in the Capital Region, Hellerup, Denmark; 2 Department of Clinical Medicine, Faculty of Health and Medical Sciences, University of Copenhagen, Copenhagen, Denmark; 3 Division of Psychotic Disorders, McLean Hospital, Belmont, MA, USA; 4 Harvard Medical School, Boston, MA, USA; 5 Mental Health Centre Amager, Copenhagen University Hospital, Copenhagen, Denmark

**Keywords:** antipsychotic medication, discontinuation, first-episode psychosis, long-term outcome, psychopharmacology, schizophrenia

## Abstract

**Introduction:**

Short-term exposure to antipsychotics has proven to be beneficial. However, naturalistic studies are lacking regarding the long-term use of antipsychotics. This study aimed to investigate changes in use of antipsychotics over 20 years after a first-episode schizophrenia.

**Methods:**

This study is part of the Danish OPUS trial (1998–2000), including 496 participants with first-episode schizophrenia. Participants were reassessed four times over 20 years. The main outcomes were days on medication, redeemed prescriptions of clozapine, psychiatric hospitalizations, and employment.

**Results:**

At the 20-year follow-up, an attrition of 71% was detected. In total, 143 out of 496 participated, with 36% (*n* = 51) in remission-of-psychotic-symptoms-off-medication. The lowest number of days on medication (mean [s.d.], 339 [538] days) was observed in this group over 20 years. Register data on redeemed antipsychotics were available for all trial participants (*n* = 416). Individuals in treatment with antipsychotics (*n* = 120) at the 20-year follow-up had spent significantly more days in treatment (5405 [1857] *v.* 1434 [1819] mean days, *p* = 0.00) and more had ever redeemed a prescription of clozapine (25% *v.* 7.8%, *p* = 0.00) than individuals who had discontinued antipsychotics (*n* = 296). Further, discontinuers had significantly higher employment at the 20-year follow-up (28.4% *v.* 12.5%, *p* = 0.00).

**Conclusion:**

In a cohort of individuals with first-episode schizophrenia, 36% were in remission-of-psychotic-symptoms-off-medication. However, high attrition was detected, potentially affecting study results by inflating results from individuals with favorable outcomes. From register data, free from attrition, approximately 30% were in treatment with antipsychotics, and 70% had discontinued antipsychotics. Individuals in treatment had the least favorable outcomes, implying greater illness severity.

## Introduction

Treatment of first-episode psychosis with antipsychotic medication has proven to be beneficial in the short term (Kishi et al., 2019). The National Institute for Health and Care Excellence guidelines suggest approximately 2 years of treatment after remission of psychotic symptoms for first-episode schizophrenia (Recommendations | Psychosis and schizophrenia in adults: prevention and management | Guidance | NICE, 2014) whereas American guidelines recommend maintaining full dose of antipsychotics (Goff et al., 2017). Overall, most clinical guidelines recommend continuing treatment with antipsychotics after remission (Hui et al., 2019). One of the main concerns with discontinuation of antipsychotic medication is the risk of psychotic relapse (Moncrieff et al., 2019) with documented higher risk of relapse among patients in remission who discontinue antipsychotic treatment compared to patients with sustained use of antipsychotics (Kishi et al., 2019). The current literature emphasizes that treatment with antipsychotics is associated with many potential side effects (Leucht et al., 2009). Still, a recent review, including studies on long-term treatment with antipsychotic medication, found that the effectiveness and efficacy of antipsychotics exceed the potential adverse side effects (Correll, Rubio, & Kane, 2018).

Few prospective cohort studies have investigated the long-term effects of antipsychotics (Gotfredsen et al., 2017; Harrow, Jobe, & Faull, 2014; Harrow et al., 2017; Kotov et al., 2017). Previous findings from the Danish OPUS study (Gotfredsen et al., 2017) revealed that approximately 30% of patients were in symptom remission and no longer in treatment with antipsychotic medication 10 years after a first episode of schizophrenia. The Suffolk County Mental Health Project (i.e. covering one of the longest follow-up studies of a modern cohort of individuals diagnosed with a first episode of psychosis) reported a significant decline in global functioning (Kotov et al., 2017) during a 20-year follow-up period as well as a remission rate of 10% in schizophrenia at the 25-year follow-up (Tramazzo et al., 2022) despite consistently high rates of treatment with antipsychotic medication over decades. Also, the 20-year follow-up of the Chicago follow-up study reported significantly better outcomes among individuals with schizophrenia not continuously treated with antipsychotics (Harrow et al., 2014, 2017). However, prospective cohort studies cannot distinguish cause and effect. Also, selection bias, reverse causation, and confounding by indication or symptom severity must be taken into consideration when interpreting findings with suggested possible long-term benefits of discontinuation of antipsychotic treatment.

This study aimed to investigate changes in the use of antipsychotic medication over 20 years among individuals diagnosed with a first-episode schizophrenia spectrum disorder; for simplicity, first-episode schizophrenia should be viewed as a subgroup of the broader term first-episode psychosis. By combining clinical interview data with longitudinal register data, we explored the number of medicated days, including initiations and discontinuations, redeemed prescriptions of clozapine, psychiatric hospitalizations, and employment status among users and those who had discontinued antipsychotic medication at year 20. Clinical characteristics, including remission rates, symptom severity, and global levels of functioning, were also reported.

## Methods

### Study population and design

This study was part of a Danish randomized controlled trial called the OPUS trial, including 496 participants with a first episode of schizophrenia. The trial was designed to investigate early intervention services compared to the available community treatment between 1998 and 2000 and described in detail in previous publications (Bertelsen et al., 2008; Thorup et al., 2005). Since the goals of this study are unrelated to the treatment assignment in the OPUS trial, the participants have been merged into one cohort. Participants were between 18 and 45 years old at the time of inclusion. They had been diagnosed with a first episode of schizophrenia spectrum disorders (F20, F22–25, F28–29) according to the International Classification of Diseases, 10th revision (ICD-10) (World Health Organization, 1993). Participants were excluded if they had received more than 3 months of treatment with antipsychotics before study inclusion. The initiation of any antipsychotic or other psychotropic medication was the decision of the treating clinician at the time of inclusion. Trained clinical staff performed assessments of the participants at 1 (Petersen et al., 2005b), 2 (Petersen et al., 2005a), 5 (Bertelsen et al., 2008), 10 (Gry Secher et al., 2015), and 20 (Hansen et al., 2023) years. At the 20-year follow-up, data were assessed from 143 individuals at the clinical interview.

### Clinical interview data

The following clinical items were used at the 1-, 2-, 5-, 10-, and 20-year follow-up interviews: the Schedules for Clinical Assessment in Neuropsychiatry (SCAN 2.1) (Wing, Sartorius, & Üstun, 1998), the Scale of Assessment of Positive Symptoms (SAPS) (Andreasen, Flaum, Swayze II, Tyrrell, & Arndt, 1990), the Scale of Assessment of Negative Symptoms (SANS) (Andreasen, 1989), and the Global Assessment of Functioning (GAF-F) (Aas, 2010). At the 10- and 20-year follow-up assessment, the Brief Assessment of Cognition in Schizophrenia (Keefe et al., 2004) and the Personal and Social Performance Scale (Tegeler & Juckel, 2007) were also assessed. Also, at baseline, pre-morbid social and academic functioning was assessed using the Pre-morbid Adjustment Scale (Brill et al., 2008), as well as the duration of untreated psychosis (DUP). Furthermore, information was gained on living conditions, parenting, educational level, employment and workability, housing and homelessness, substance abuse, and current use of antipsychotic medication (incl. generic name of antipsychotic, dosage, adherence, and administration route).

### Registry data

The Danish National Prescription Registry (Pottegård et al., 2017) contains individual-level data on all redeemed prescriptions of antipsychotic medication with the following ACT codes: N05Ax except N05AN Lithium.

The Danish Psychiatric Central Register (Mors, Perto, & Mortensen, 2011) holds information on psychiatric hospitalization and outpatient contacts.

Employment status was available from the DREAM database (Statistics Denmark, 2014).

### Definition of outcomes in clinical interview data

Use and no use of antipsychotic medication was based on self-report by the participants at baseline, 1, 2, 5, 10, and 20 years. Users of antipsychotics were defined as consumers of oral antipsychotic medication or long-acting injectable medication at the time of follow-up. No use of antipsychotic medication was defined as no consumption or use of long-acting injectables at the time of follow-up.

Remission of psychotic and negative symptoms was defined as a score of 2 or less on all items on either SAPS or SANS, respectively, for at least 6 months in accordance with the Remission in Schizophrenia Working Group (Andreasen et al., 2005). Remission of psychotic symptoms was the mean score between the global ratings for hallucinations and delusions. Remission of negative symptoms was the mean score of the four global negative domains, excluding the attention domain.

Clinical recovery was defined as no psychotic episode, no psychiatric hospitalizations, and no supported housing or accommodation for 2 consecutive years before follow-up and currently studying or working and a GAF-F score of 60 or above, as reported in previous publications (Austin et al., 2013; Hansen et al., 2023).

### Statistical analysis

The register-based analyses included all individuals until death, emigration, December 31, 2021, or whichever came first. Variables were tested individually using a *t* test if the variable was continuous or ordinal or a Chi-squared test if the variable was categorical. Non-parametric tests were used to test differences in continuous data with skewed distributions (i.e. Mann–Whitney's *U* and Kruskal–Wallis test) including DUP, medicated days, initiation and discontinuations, days of psychiatric hospitalizations, and outpatient contacts.

We did not have data on antipsychotic medication during psychiatric hospitalization or on patients receiving free-of-charge antipsychotic medication.

Medicated days with antipsychotics were defined as all redeemed antipsychotics with at least 30 defined daily doses (DDD). We determined daily whether a person was on medication for the entire period from inclusion to the end of follow-up. Each redeemed prescription was considered active for a period of DDD × 1.15 + 30 days, as described (Tiihonen, 2012) and used (Stürup et al., 2022) in previous studies. Regardless of prescriptions, patients were considered on medication on days when they were admitted to a psychiatric inpatient unit since individuals admitted to a psychiatric hospital were assumed to be treated with antipsychotic medication during admission. We counted the number of times a person went from being off medication to on medication (number of initiations) and the number of times a person went from being on medication to off medication (number of discontinuations).

‘Ever on clozapine’ was defined as individuals who redeemed a clozapine prescription through 20 years of follow-up.

To report on long-term outcomes between use and no use of antipsychotic medication, we divided the participants of the 20-year follow-up into four groups: remission-of-psychotic-symptoms-off-medication, remission-of-psychotic-symptoms-on-medication, non-remission-of-psychotic-symptoms-off-medication, and non-remission-of-psychotic-symptoms-on-medication (Table 1). To be considered in remission of psychotic symptoms, the global scores for hallucinations, delusions, bizarre behavior, and thought disorder should be 2 or less for six consecutive months on the SAPS scale.

**Table 1.** Clinical characteristics based on remission of psychotic symptom status and use of antipsychotic medication at year 20

	All, *n* = 143	Remission-of-psychotic-symptom-off-medication^a,b^ (*n* = 51) 36%	Remission-of-psychotic-symptom-on-medication^a,b^ (*n* = 42) 29%	Non-remission-of-psychotic-symptom-off-medication^a^ (*n* = 13) 9%	Non-remission-of-psychotic-symptom-on-medication^a^ (*n* = 37) 26%	*p* Value
Sociodemographic, *n* (%), 20-year follow-up						
Female	75 (52.4)	32 (62.7)	19 (45.2)	3 (23.1)	21 (56.8)	**0.050**
Age, mean (s.d.)	46.15 (5.52)	45.31 (5.70)	46.95 (5.45)	44.62 (4.37)	46.92 (5.64)	0.289
Independent living	133 (93.0)	49 (96.0)	39 (92.9)	12 (92.3)	33 (89.2)	0.664
In a relationship	61 (42.7)	29 (56.9)	16 (38.1)	2 (15.4)	14 (37.8)	**0.031**
Living alone	74 (51.7)	13 (25.5)	27 (64.3)	8 (61.5)	26 (70.2)	**0.000**
Living with children	16 (11.2)	13 (25.5)	0 (0)	1 (7.7)	2 (5.4)	**0.000**
Living with a partner with/without children	40 (28.0)	23 (45.1)	11 (26.2)	1 (7.7)	5 (13.5)	**0.000**
Living with parents/friends	8 (5.6)	2 (3.9)	2 (4.8)	3 (23.1)	2 (5.4)	**0.000**
Living in supported housing	4 (2.8)	1 (2.0)	1 (2.4)	0 (0)	2 (5.4)	**0.000**
Working or studying	40 (28.0)	25 (49.0)	9 (21.4)	4 (30.8)	2 (5.4)	**0.000**
Educational level above primary school (baseline)	56 (39.2)	16 (31.4)	23 (54.8)	6 (46.2)	11 (29.7)	0.065
Has become a parent	67 (46.9)	36 (70.6)	13 (31.0)	4 (30.8)	14 (37.8)	**0.000**
Premorbid functioning, mean (s.d.)						
PAS, mean (s.d.), Social adaptation	0.72 (0.57)	0.70 (0.62)	0.66 (0.54)	0.54 (0.40)	0.88 (0.57)	0.212
PAS, mean (s.d.), Academic adaptation	0.96 (0.63)	0.96 (0.70)	0.88 (0.61)	0.80 (0.50)	1.11 (0.58)	0.294
DUP (in weeks), median (range)						
DUP	53.00 (0–1037.86)	42 (0–841.29)	40.36 (0–836.43)	98.71 (0–1036.43)	72.00 (0–822.71)	0.391
Psychopathology, mean (s.d.), 20-year follow-up						
SANS (all items)	1.58 (1.21)	0.80 (0.93)	1.58 (0.97)	2.02 (1.18)	2.45 (1.15)	**0.000**
SAPS (hallucinations + delusions)	1.17 (1.50)	0.17 (0.51)	0.48 (0.74)	2.42 (1.08)	2.92 (1.36)	**0.000**
Remission and clinical recovery, *n* (%)						
Remission of negative symptoms^c^	66 (46.2)	35 (68.6)	19 (45.2)	4 (30.8)	8 (21.6)	**0.000**
Clinical recovery^d^	24 (16.8)	18 (35.3)	6 (14.3)	0 (0)	0 (0)	**0.000**
Early responders of treatment^e^	39 (30)	9 (17.0)	16 (38.0)	2 (15.3)	12 (32.4)	0.068
Cognition, mean (s.d.), 20-year follow-up (*Z*-score calculated from a population including F21)						
Brief assessment of cognition in schizophrenia, total *z*-score reported in s.d.	0.06 (0.63)	0.32 (0.54)	−0.03 (0.64)	0.36 (0.50)	−0.40 (0.55)	**0.000**
Social and global functioning, mean (s.d.), 20-year follow-up						
GAF-F	56.54 (15.67)	67.72 (13.61)	56.45 (11.60)	49.77 (12.86)	43.89 (12.12)	**0.000**
Personal and social performance scale score	56.65 (15.88)	68.10 (12.93)	56.48 (11.81)	50.00 (13.48)	43.73 (13.10)	**0.000**
Use of antipsychotic medication, *n* (%), *N* (total number of participants for each participation year)						
1 year follow-up (*N* = 379)	88 (61.5)	22 (43.1)	31 (73.8)	7 (53.8)	28 (75.7)	**0.004**
2 year follow-up (*N* = 334)	81 (56.6)	19 (37.3)	26 (61.9)	5 (38.5)	31 (83.8)	**0.000**
5 year follow-up (*N* = 266)	60 (42.0)	8 (15.7)	19 (45.2)	5 (38.5)	28 (75.7)	**0.000**
10 year follow-up (*N* = 303)	67 (46.9)	4 (7.8)	28 (66.7)	3 (23.1)	32 (86.5)	**0.000**
Currently in treatment with clozapine	10 (7.0)	0 (0)	5 (11.9)	0 ()	5 (13.5)	**0.032**
Diagnoses, ICD-10, *n* (%), baseline						
Diagnosis of substance abuse	41 (28.7)	13 (25.5)	14 (33.4)	4 (30.7)	10 (27.0)	0.855
Diagnosis of substance abuse (20-year follow-up)	22 (15.4)	3 (5.9)	6 (14.3)	4 (30.7)	9 (24.3)	**0.037**
Schizophrenia (F20)	112 (78.3)	38 (74.5)	31 (73.8)	11 (84.6)	32 (86.5)	0.441
Others (F22–F25, F28–F29)	31 (21.7)	13 (25.5)	11 (26.2)	2 (15.4)	5 (13.5)	0.441
OPUS treatment	64 (44.8)	22 (43.1)	17 (40.5)	7 (53.8)	18 (48.6)	**0.031**
Longitudinal register-based data, median (range)						
Ever on clozapine, *n* (%)	22 (15.4)	2 (3.9)	7 (16.7)	0 (0)	13 (35.1)	**0.001**
Number of medicated days with antipsychotics	1521 (0–8054) 33%	74 (0–2506) 5%	4322 (0–7532) 45%	58 (0–3494) 10%	5507 (140–8054) 62%	**0.001**
Number of discontinuations	9 (0–59)	2.00 (0–18)	19.50 (0–59)	5 (0–22)	21.00 (2–47)	**0.001**
Number of initiations	8 (0–58)	1.00 (0–18)	19.00 (0–58)	4.00 (0–22)	20.00 (2–46)	**0.001**
Days of psychiatric hospitalizations between year 10 and 20	0 (0–1492)	0 (0–81)	0 (0–1492)	0 (0–446)	67 (0–549)	**0.001**

*p*-values less than 0.05 are in bold. GAF-F, Global Assessment of Functioning Scale; PAS, Premorbid Adjustment Scale; DUP, Duration of Untreated Psychosis; SAPS, Scale for Assessment for Positive Symptoms; SANS, Scale for Assessment of Negative Symptoms; ICD-10, International Statistical Classification of Diseases, Tenth Revision; OPUS, two-year intensive early intervention program.

a Remission-of-psychotic-symptoms-off-medication, remission-of-psychotic-symptoms-on-medication, non-remission-of-psychotic-symptom-off-medication, and non-remission-psychotic-symptoms-on- medication were based on all items on Scale for Assessment for Positive Symptoms (SAPS) and use of antipsychotic medication. To be considered non-psychotic and in remission, the global scores for hallucinations, delusions, bizarre behavior, and thought disorder should be two or less for six consecutive months in accordance with the Remission in Schizophrenia Working Group.

^b^Remission of psychotic symptoms: defined as a score of 2 or less on all items on the Scale for Assessment for Positive Symptoms (SAPS) for at least six months in accordance with the Remission in Schizophrenia Working Group.

c Remission of negative symptoms: defined as a score of 2 or less on all items on the Scale for Assessment of Negative Symptoms (SANS), excluding the attention domain, for at least 6 months in accordance with the Remission in Schizophrenia Working Group.

d Clinical recovery: defined as no psychotic episode, no psychiatric hospitalizations, and no supported accommodation for two consecutive years before follow-up, currently studying or working, and a GAF-F score of 60 or more.

e Early responders of treatment: defined as individuals with a score above two on the items of hallucinations and delusions on SAPS at baseline, following a score of 2 or less at 1-year follow-up and in treatment with antipsychotics.

In the register-based analysis, we divided the entire sample (Table 2) and the clinically assessed sample (Table 3) into discontinuation and use of antipsychotics at year 20 based on the abovementioned algorithm.

**Table 2.** Longitudinal register-based data and clinical baseline characteristics among the entire sample of 416 individuals divided between current discontinuation or use of antipsychotic medication at year 20

	All, *n* = 416	Discontinuation *n* = 296 (71%)	Use of antipsychotics *n* = 120 (29%)	*p* Value
Sociodemographic variables at baseline, *n* (%)				
Female	185 (44.5)	137 (46.3)	48 (40.0)	0.243
Age at inclusion, mean (s.d.)	27 (6)	27 (7)	27 (6)	0.965
Independent living	317 (76.2)	227 (76.7)	90 (75.0)	0.273
In a relationship	101 (24.3)	78 (26.4)	23 (19.2)	0.107
married	26 (6.3)	19 (6.4)	7 (5.8)	0.829
Being a parent	61 (14.7)	44 (14.9)	17 (14.2)	0.814
Working or studying	126 (30.3)	89 (30.0)	37(30.8)	0.878
Clinical variables at baseline, mean (s.d.)				
DUP in weeks, median (range)	45.00 (0–1039.43)	43.29 (0–1039.43)	46.00 (0–852.86)	0.506
SAPS	3.02 (1.33)	2.99 (1.31)	3.03 (1.37)	0.329
SANS	2.16 (1.17)	2.13 (1.17)	2.24 (1.17)	0.383
GAF-F	30.84 (13.46)	38.89 (13.06)	41.62 (13.56)	0.064
Diagnoses, ICD-10, *n* (%), baseline				
Substance abuse	107 (25.5)	81 (27.4)	26 (21.7)	0.228
Schizophrenia (F20)	321 (77.2)	225 (76.0)	96 (80.0)	0.380
Others (F22–F29)	98 (23.6)	71 (24.0)	27 (22.5)	
OPUS-treatment	203 (48.8)	139 (47.0)	64 (53.4)	0.218
Longitudinal register-based data with information on all participants (without attrition), median (range)				
Ever on clozapine *n* (%)	53 (12.7)	23 (7.8)	30 (25.0)	**0.000**
Number of days medicated	1452 (0–8090) 33%	522 (0–8090) 17%	5614 (684–8058) 65%	**0.001**
Number of initiations	8 (0–75)	5 (0–66)	19 (2–75)	**0.001**
Number of discontinuations	9 (0–76)	5 (0–65)	20 (2–76)	**0.001**
Days of psychiatric hospitalizations between year 10 and 20	0 (0–3653)	0 (0–3605)	9 (0–3653)	**0.001**
Outpatient contacts years 10–20	13 (0–1285)	3 (0–1285)	46 (0–509)	**0.001**
Supported housing year 20, *n* (%)	11 (2.6)	7 (2.4)	4 (3.4)	0.554
Work^a^ *n* (%)	54 (13.0)	49 (16.6)	3 (2.5)	**0.000**
Any work^b^ *n* (%)	105 (25.2)	84 (28.4)	15 (12.5)	**0.000**

*p*-values less than 0.05 are in bold. GAF-F, Global Assessment of Functioning Scale; PAS, Premorbid Adjustment Scale; DUP, Duration of Untreated Psychosis; SAPS, scale for Assessment for Positive Symptoms; SANS, Scale for Assessment of Negative Symptoms; ICD-10, International Statistical Classification of Diseases, Tenth Revision, OPUS, two-year intensive early intervention program.

a In full-time employment without supported employment benefits 50% of the past year before the 20-year follow-up.

b Full- or part-time employment (with/without supported employment benefits) 50% of the past year before the 20-year follow-up.

**Table 3.** Longitudinal register-based data among 143 individuals in current discontinuation or use of antipsychotic medication at year 20

	All, *n* = 143	Discontinuation *n* = 64 (45%)	Use of antipsychotics *n* = 79 (55%)	*p* Value
Longitudinal register-based data with information on all participants (without attrition), median (range)				
Ever on clozapine, *n* (%)	22 (15.4%)	2 (3.1%)	20 (25.3%)	**0.001**
Number of days medicated	1521 (0–8054) 32%	97 (0–3494) 6%	4585 (0.8054) 53%	**0.000**
Number of initiations	8 (0–58)	1 (0–22)	20 (0–58)	**0.000**
Number of discontinuations	9 (0–59)	2 (0–22)	20 (0–59)	**0.000**
Days of psychiatric hospitalizations between year 10 and 20	0 (0–1492)	0 (0–446)	20 (0–1492)	**0.000**
Outpatient contacts years 10–20	17 (0–647)	0 (0–162)	56 (0–647)	**0.000**
Supported housing year 20, *n* (%)	1 (0.7%)	0 (0.0%)	1 (1.3%)	0.347
Work^a^, *n* (%)	27 (18.9%)	22 (34.4%)	5 (6.3%)	**0.001**
Any work^b^, *n* (%)	48 (33.6%)	29 (45.3%)	19 (24.1%)	**0.007**

*p*-values less than 0.05 are in bold.

a In full-time employment without supported employment benefits 50% of the past year before the 20-year follow-up.

b Full- or part-time employment (with/without supported employment benefits) 50% of the past year before the 20-year follow-up.

**Figure 1.**
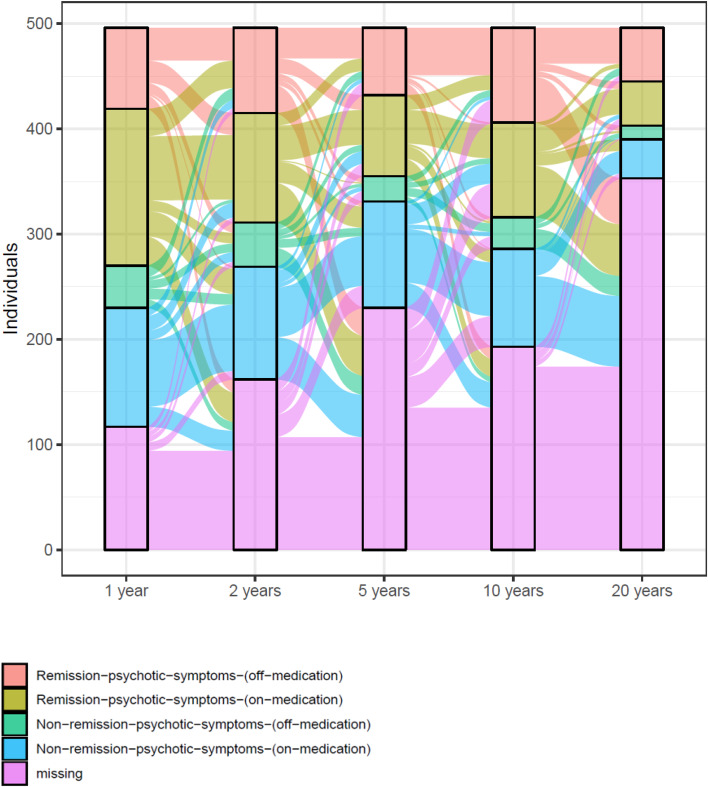
Proportional flow between groups of remission of psychotic symptoms on/off antipsychotic medication through a period of 20 years among 496 individuals with a first episode of schizophrenia. Remission-of-psychotic-symptoms-off-medication, remission-of-psychotic-symptoms-on-medication, non-remission-of-psychotic-symptom-off-medication, and non-remission-of-psychotic-symptom-on-medication were based on all items in the SAPS interview and intake of antipsychotic medication. To be considered non-psychotic, the global scores for hallucinations, delusions, bizarre behavior, and thought disorder should be 2 or less for six consecutive months in accordance with the Remission in Schizophrenia Working Group.

We used a Sankey diagram to illustrate the proportional flow between the four groups of remission of psychotic symptoms on- or off-antipsychotic medication over a period of 20 years (Fig. 1). Also, the proportional flow between clinical reports of use and no use of antipsychotic medication over 20 years among 496 individuals is illustrated in eFig. 2 in the online Supplementary material.

All analyses were performed using IBM SPSS statistical software versions 25 and 27 (SPSS Inc.). Two-sided *p* values were statistically significant at less than 0.05. The Sankey diagram was created using R-statistical software version 4.2.2 with the following packages: data.table (version 1.14.8; Dowle M, Srinivasan A, 2023), ggalluvial (version 0.12.5; Brunson JC, Read QD, 2023), and ggplot2 (version 3.4.3; Wickham H, 2016).

### Ethical standards

The authors assert that all procedures contributing to the OPUS trial have been approved by the Regional Ethical Scientific Committee (Protocol no. 17023873) and by the Danish data protection agency (RHP-2017-047, I-Suite no.: 05855) complying with the ethical standards of the relevant national and institutional committees on human experimentation and with the Helsinki Declaration of 1975, as revised in 2008. Furthermore, the trial has been registered at ClinicalTrials.gov NCT00157313.

## Results

### Clinical characteristics of the study population at year 20

At the 20-year follow-up, 143 individuals participated with a mean age of 46.17 years (range 38–64 years); 78% were diagnosed with schizophrenia (F20), whereas 22% were diagnosed with another F2 diagnosis within the schizophrenia spectrum (F22–25, F28–29) (Table 1). These numbers mirror those of the initial cohort, with a majority of 77% of individuals qualifying for a diagnosis of schizophrenia and a minority of 24% qualifying for another schizophrenia spectrum disorder (Table 2). No differential attrition between diagnoses among participants and non-participants at the 20-year follow-up was found (see online Supplementary material eTable 1 for drop-out analyses). Furthermore, a flowchart of study participation through 20 years is illustrated in eFig. 1 in the online Supplementary material. Still, participants at the 20-year follow-up were younger (*p* = 0.01), and more females participated (*p* = 0.00) than non-participants. Non-participants had higher scores of negative (*p* = 0.01) and disorganized symptom dimensions (*p* = 0.02) and lower scores on the GAF-F scale (*p* = 0.00) and on the personal and social performance scale (*p* = 0.04), and fewer had completed high school (*p* = 0.03) compared to participants. See online Supplementary eTable 1 for drop-out analysis. Also, an additional drop-out analysis found the same trends in attrition investigating 10-year clinical data among participants and non-participants at the 20-year follow-up (see eTable 2 in the online Supplementary material).

Of those who were in remission-of-psychotic-symptoms-off-medication (*n* = 51), 29% were also in remission of psychotic symptoms and off-antipsychotic medication at year 1, 33% at year 2, 53% at year 5, and 67% at year 10. Also, Fig. 1 illustrates the dynamic interplay of antipsychotic medication status and symptom remission across the five follow-up assessments. Overall, the data resemble the fluctuating use of antipsychotics as well as shifts in and out of symptom remission over 20 years, reflecting the reality of real-world patients.

At the 20-year follow-up, participants in remission-of-psychotic-symptoms-off-medication at year 20 (*n* = 51) had the lowest percentage of participants diagnosed with schizophrenia (74.5%) and the highest percentage of participants diagnosed with another schizophrenia spectrum disorder (25.5%) (Table 1). Also, more participants had become parents (70.6%) and were studying or working (49.0%) in year 20 compared to the other groups (Table 1). Furthermore, this group had the highest percentage of participants in remission of negative symptoms (68.6%) and in clinical recovery (35.3%) compared to the other groups (Table 1). Also, this group had the highest score on the personal and social performance scale, with a mean (s.d.) of 68.10 (12.93) compared to all other groups (Table 1). The participants had reported the lowest percentages of use of antipsychotics at all follow-up points, with 43% at year 1, 37.3% at year 2, 15.7% at year 5, and 7.8% using antipsychotics at year 10 compared to the rest of the groups (Table 1).

Participants in remission-of-psychotic-symptoms-on-medication at year 20 (*n* = 42) had 45.2% of participants in remission of negative symptoms, and 14.3% of individuals had obtained clinical recovery (Table 1). At the previous follow-ups, the participants had reported high percentages of use of antipsychotics ranging from 61.9% to 73.8%, with 11.5% in current treatment with clozapine (Table 1).

Participants in non-remission-of-psychotic-symptoms-off-medication at year 20 (*n* = 13) had a high percentage of participants diagnosed with schizophrenia (84.6%) and a low percentage of participants diagnosed with another schizophrenia spectrum disorder (15.4%) (Table 1). Also, few participants were in remission of negative symptoms (30.8%) (Table 1). At the previous follow-ups (1-, 2-, 5-, and 10-year follow-ups), low percentages of use of antipsychotic medication ranging from 23.1% to 53.8% were reported (Table 1).

Participants in non-remission-of-psychotic-symptoms-on-medication at year 20 (*n* = 37) had the highest percentage of participants diagnosed with schizophrenia (86.5%) and the lowest percentage of participants diagnosed with another schizophrenia spectrum disorder (13.5%) (Table 1). Also, the lowest percentage of participants in remission of negative symptoms (21.6%) was found in this group. They also had the lowest score on the personal and social performance scale, with a mean (s.d.) of 43.73 (13.10) (Table 1). At the previous follow-ups (1-, 2-, 5-, and 10-year follow-up), more participants were treated with antipsychotic medication ranging from 75.7% to 86.5% compared to the other groups, and currently, 13.5% were under treatment with clozapine (Table 1).

### Register-based outcomes for 143 individuals participating in the 20-year follow-up

**Figure 2.**
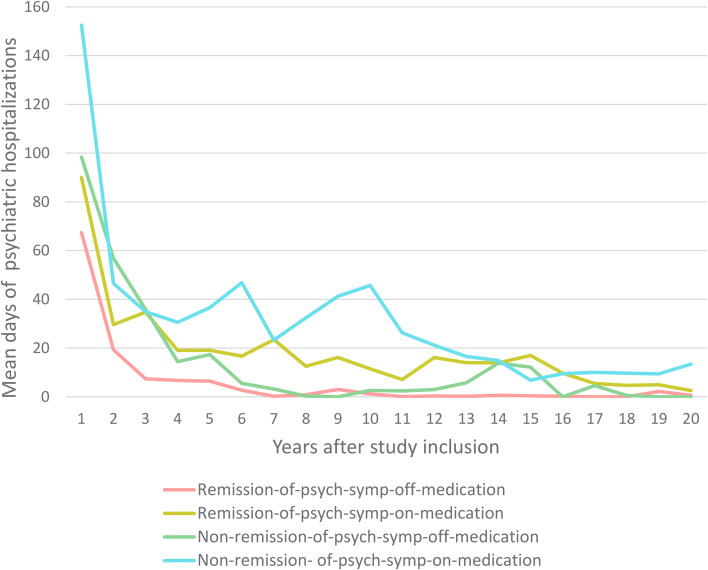
Days of psychiatric hospitalizations over 20 years. Remission-of-psychotic-symptoms-off-medication, remission-of-psychotic-symptoms-on-medication, non-remission-of-psychotic-symptom-off-medication, and non-remission-of-psychotic-symptom-on-medication were based on all items in the SAPS interview and intake of antipsychotic medication. To be considered non-psychotic, the global scores for hallucinations, delusions, bizarre behavior, and thought disorder should be 2 or less for six consecutive months in accordance with the Remission in Schizophrenia Working Group.

When using the Danish longitudinal register-based data with information in between follow-up points, the participants in remission-of-psychotic-symptoms-off-medication at 20 years (*n* = 51) had the lowest number of days on medication (median [range], 74 days [0–2506]) with only 5% of observed days spent in treatment with antipsychotics over 20 years. The median (range) of initiations and discontinuations with antipsychotic medication were 1 (0–18) and 2 (0–18), respectively. Also, 3.9% (*n* = 2) of participants had ever redeemed a clozapine prescription (Table 1). Furthermore, the group had the lowest number of psychiatric hospitalizations over 20 years of follow-up compared to the other groups (Fig. 2).

Participants in remission-of-psychotic-symptoms-on-medication (*n* = 42) had a high number of days on medication (median [range], 4322 [0–7532] days) with 45% of observed days spent in treatment with antipsychotics over 20 years. Furthermore, an almost equal median (range) of 19.00 (0–58) initiations and 19.50 (0–59) discontinuations of antipsychotics over 20 years was found (Table 1). Also, 16.7% (*n* = 7) of participants had ever redeemed a clozapine prescription (Table 1).

Participants in non-remission-of-psychotic-symptoms-off-medication (*n* = 13) had no redeemed prescriptions of clozapine over 20 years (Table 1). They had a median (range) of 158 (0–3494) days on antipsychotics, with 10% of the time spent in treatment. An almost equal median (range) number of 4.00 (0–22) initiations and 5 (0–22) discontinuations over 20 years was found (Table 1).

The participants in the non-remission-of-psychotic-symptoms-on-medication had the highest number of days on medication (median [range], 5507 [140–8054] days) with 62% of observed days spent in treatment with antipsychotics over 20 years. A median (range) of 20 (2–46) initiations and 21 (2–47) discontinuations over 20 years was found (Table 1). Furthermore, the highest percentage of 35.1% (*n* = 13) having ever redeemed a clozapine prescription was found compared to the other groups (Table 1). Days of psychiatric hospitalizations were also higher for this group than for all the other groups in comparison over 20 years (Fig. 2).

### Register-based outcomes (with no attrition) for the entire study population

In the register-based analysis, 296 (71%) participants had discontinued, and 120 (29%) were users of antipsychotic medication in year 20 (Table 2). No significant differences were found between the groups on various baseline sociodemographic and clinical variables, including sex, age, living conditions diagnoses, DUP, GAF-F, and psychotic and negative symptoms dimensions (Table 2). On register-based outcomes, a significant difference was found for ever having redeemed a prescription of clozapine, with 23 individuals (7.8%) in the discontinuation group and 30 individuals (25%) in the user of antipsychotics group (*p* = 0.00). Furthermore, significant differences were found on medicated days between the two groups with 17% of observed days spent in treatment (median [range], 522 [0–8090] days) in the discontinuation group compared to 65% (median [range], 5614 [684–8058] days) in the user of antipsychotics group over 20 years (*p* = 0.00) (Table 2). Also, significant differences in psychiatric hospitalizations and outpatient contacts were found, with a median (range) of 0 (0–3605) *v.* 9 (0–3653) days of psychiatric hospitalizations and 3 (0–1285) *v.* 46 (0–509) days of outpatient contacts between the discontinue group compared to the antipsychotics group, respectively (*p* = 0.00) (Table 2). When investigating employment, significantly fewer of the users of the antipsychotics group (12.5% *v.* 28.4%) were in full- or part-time employment (with/without supported employment benefits) 50% of the past year before the 20-year follow-up compared to the discontinuation group (*p* = 0.00) (Table 2).

When dividing the same clinical sample (*n* = 143) into discontinuation (*n* = 64) and use of antipsychotics (*n* = 79) at year 20, the same significant trends in register-based data were evident among users of antipsychotics (i.e. with higher number of medicated days, more days in psychiatric hospitals, more outpatient contacts, and less in full and part-time employment) compared to individuals who had discontinued antipsychotics at year 20 (Table 3).

## Discussion

In this 20-year follow-up of the original OPUS trial, 143 individuals participated in the clinical interview (i.e. 78% with schizophrenia and 22% with another schizophrenia spectrum disorder excluding schizotypal disorders). To further investigate changes in the use of antipsychotics, we divided the participants into four groups based on remission of psychotic symptoms status and use of antipsychotics.

Overall, 36% of the participants were in remission-of-psychotic-symptoms-off-medication. This group had more favorable outcomes regarding living conditions, psychopathology, clinical recovery, and social and functional scores at the 20-year follow-up than all other groups. In the 10-year follow-up of the OPUS trial, female sex, higher global functioning scores, employment, and absence of substance abuse were predictors of being in remission-off-antipsychotics (Wils et al., 2017). However, as of now, there are no consistent predictors of successful discontinuation of antipsychotics among individuals diagnosed with schizophrenia (Schoretsanitis et al., 2022).

In this 20-year follow-up study, we found no significant differences between baseline characteristics, including premorbid social- and academic functioning, diagnoses, DUP, substance abuse, and educational level above primary school, between those in treatment and those who had discontinued antipsychotics at year 20. This might imply that it is difficult to predict or foresee long-term outcomes of the use of antipsychotics based on baseline variables. Still, in line with the general literature, DUP is associated with more severe symptoms at follow-up, and this is also reflected in our results where the two non-remission groups have longer duration of untreated illness than the two groups in remission (Penttilä et al., 2014).

Even though we could not find baseline differences between those in treatment and those who had discontinued antipsychotics at year 20, a Finnish register-based cohort study investigating discontinuation of antipsychotics among first-episode schizophrenia did not find relapses to decrease among individuals discontinuing antipsychotic medication (Tiihonen, Tanskanen, & Taipale, 2018). Instead, they found increased survival among users of antipsychotics followed for 20 years.

In our study we found a higher number of days medicated over 20 years in the two groups on-antipsychotics, regardless of the remission status. Also, notably higher numbers of initiations and discontinuation of antipsychotics were found in the two groups on-antipsychotics compared to those off-antipsychotics. The groups on-antipsychotics also had higher percentages of ever having redeemed a prescription of clozapine, and they also tended to report higher numbers of use of antipsychotics at every follow-up point compared to groups off-antipsychotics.

Notably, 13 (35.1%) from the non-remission-of-psychotic-symptoms-on-medication had ever redeemed a prescription of clozapine, and only 5 (13.5%) were in current treatment with clozapine. Since clozapine is recommended for treatment-resistant schizophrenia, it is conspicuous why so markedly few individuals have ever redeemed a prescription of clozapine. There can be several reasons for not continuing clozapine treatment (given the high rates of side effects and necessary compliance with regular blood samples). Still, treatment-resistant patients should at least have one trial of clozapine as it can have a major impact on the illness burden. Our findings question whether individuals in non-remission of psychotic symptoms are offered the recommended medical treatment.

Another interesting finding concerns psychiatric hospitalizations, with groups on-antipsychotic medication on average spending more days in psychiatric hospitals from year 3 to year 20 compared to groups off-antipsychotic medication, implying worse outcomes.

At the 20-year follow-up of the Suffolk County Mental Health cohort, a significant decline in global functioning was also detected among 175 individuals with schizophrenia spectrum disorders despite a consistently high rate of use of antipsychotic medication (Kotov et al., 2017). We can only speculate why the results from Suffolk County are so different from those of the 20-year follow-up of the Danish OPUS trial. The duration of operational diagnostic criteria of schizophrenia differs from one month in ICD-10 to 6 months in DSM-V (Valle, 2020), potentially leading to more chronic illness stages in the Suffolk County cohort. Perhaps, also, health care systems and the setting these patients are a part of could explain some of the differences observed. Also, the majority of the Suffolk County Mental Health cohort consists of men, and from the literature, we know that, on average, men have worse outcomes than women.

Another notable finding is that cognitive function was higher in medication-free groups both in remission and non-remission of symptoms compared to the groups still using antipsychotics. This could suggest that in later stages of illness, antipsychotic medication is more strongly associated with cognitive difficulties than the level of psychotic symptoms. Also, another result worth noticing is that more participants who had a substance diagnosis were also in non-remission of psychotic symptoms, independently of whether they had discontinued use of antipsychotics or not. This is in line with research showing substance as an independent risk factor for poor outcomes in schizophrenia.

Using the Danish prescription register with data on redeemed antipsychotics on all trial participants (*n* = 416) between follow-up points, we found significant differences between the two groups. Those in treatment with antipsychotics at the 20-year follow-up spent almost four times as many days in treatment and three times as many had redeemed a prescription of clozapine than those who have discontinued treatment with antipsychotics over 20 years. Also, those who were in treatment with antipsychotics had doubled the amount of both initiations and discontinuations of antipsychotics, as did those in the discontinue group. Further, those in the discontinued group had twice as high employment the past year before the 20-year follow-up compared to those in treatment with antipsychotics.

All the findings mentioned above could be a matter of cause and not effect since observational studies are vulnerable to confounding. Individuals with lower illness severity are likely to discontinue antipsychotic treatment and, therefore, naturally have better outcomes, including increased functioning. In this scenario, the baseline characteristics of individuals with high or low severity may be similar. Still, differences emerge over the course of years that determine which patients have a more severe course of illness (and therefore remain on medications) and which have a less severe course (and therefore can come off medications). Also, observational studies have the limitations of selection bias, reverse causality, and confounding by diagnosis and severity.

### Limitations

In general, all redeemed prescription of antipsychotic medication is registered in the Danish National Prescription Registry, including long-acting injectables. Still, the registers do not hold information on antipsychotic medication during psychiatric hospitalization or on patients receiving free-of-charge antipsychotic medication; therefore, our findings on the use of antipsychotics should be considered conservative, and the true number of days on antipsychotics might be underestimated in this cohort. For example, the percentage of discontinuation *v.* use of antipsychotics in the original 416 sample was 71% *v.* 29% based on register-based data (Table 3). However, the self-reported percentage of discontinuation *v.* use of antipsychotics among the 143 individuals clinically assessed in the 20-year follow-up was 44% *v.* 56% (see medication status among the four groups in Table 1). This comparison between self-reported data on antipsychotics and register-based data clearly illustrates the potential lack of 20% redeemed prescriptions in the Danish prescription register, which might be explained by ‘free of charge-medicine’. On the other hand, we did include days in psychiatric hospitalization as days on medication and thus generated more validity in our registered data on antipsychotics. However, at the 20-year follow-up, 52 out of 64 individuals provided information on the decision-making about discontinuation. A total of 72% (*n* = 39) responded that it was their own choice to discontinue, 19% (*n* = 10) responded that their physician suggested discontinuation, and 6% (*n* = 3) responded that it was suggested by another person (i.e. spouse, partner, friend, etc.).

In addition, differences between the groups could either be cause or effect since observational studies are affected by confounding by indication (e.g. individuals with psychotic symptoms are on antipsychotics rather than individuals on antipsychotic medication have psychotic symptoms).

Furthermore, we are aware of the possible overestimation of individuals with a favorable outcome from the clinical interview data in our study (Hansen et al., 2023). Also, when conducting drop-out analyses, the same trends in drop-out were found using baseline and 10-year clinical data, with more individuals with favorable outcomes participating at the 20-year follow-up (i.e. more females, better global functioning, fewer symptoms, and higher percentages of individuals in remission of psychotic symptoms). This shows that the numbers of individuals with favorable outcomes are potentially inflated due to higher dropout rates among those with unfavorable outcomes. Likewise, from the illustration of proportional flow between groups (Fig. 1), the highest drop-out was found among those in non-remission, thereby potentially inflating the results from those in remission off of medication. Also, results should be interpreted cautiously due to multiple cross-sectional comparisons. Still, we find these results relevant since the associations of use of antipsychotics and remission status are understudied in long-term follow-ups among individuals with schizophrenia. Therefore, we would rather report few false positives than miss out on important findings. Finally, no data on symptom remission were available in between clinical follow-ups.

## Conclusions

Overall, 36% of participants were in remission-of-psychotic-symptoms-off-medication at 20-years follow-up. This group also had more favorable outcomes regarding living conditions, psychopathology, clinical recovery, and social and functional scores at the 20-year follow-up than all other groups. Still, the highest drop-out was found among the groups in non-remission, thereby potentially inflating the results from those in remission off medication. Register data for the entire study population, free from attrition, found users of antipsychotics to spend significantly more days in treatment, redeemed more prescriptions of clozapine, and were less likely to be employed than those who had discontinued antipsychotics at year 20, implying greater illness severity among individuals in treatment with antipsychotics.

## Supporting information

Hansen et al. supplementary material Hansen et al. supplementary material

## Supplementary material

The supplementary material for this article can be found at https://doi.org/10.1017/S0033291724002678.
