# Supplementary material for: Use and discontinuation of antipsychotic medication in 20 years following a first episode of schizophrenia: results from the OPUS trial
Source: Psychol Med. 2024 Nov 18;54(15):4374–84. doi: 10.1017/S0033291724002678 (PMC11650151; doi:10.1017/S0033291724002678)
Supplement: Hansen et al. supplementary material [file S0033291724002678sup001.docx]

**Supplementary content**

Hansen H.G., Speyer H, et al. Use and Discontinuation of Antipsychotic Medication in 20 Years following a First Episode of Schizophrenia- Results from the OPUS Trial

**eTable 1: Baseline characteristics among participants and non-participants at 20-year follow-up** ......................**2**

**eTable 2: 10-year characteristics among participants and non-participants at the 20-year follow-up** ……...…..**3**

**eFigure 1:** **Flow-chart of participation**…………..…………………...……………….................................................**4**

**eFigure 2:** **Proportional flow between users and no use of antipsychotic medication through 20 years** ...............**5**

| **eTable 1. Baseline characteristics among participants and non-participants at 20-year follow-up** | | | | |
| --- | --- | --- | --- | --- |
|  | All,  n=496 | Participants,  n=143 | Non-participants,  n=353 | P value |
| *Sociodemographics, n (%)* | | | | |
| Age, mean (SD) | 26.80 (6.50) | 25.62 (5.6) | 27.27 (6.74) | **.005** |
| Sex, Female | 206 (41.5) | 75 (52.4) | 131 (37.1) | **.003** |
| Studying or working | 142 (28.6) | 43 (30.0) | 99 (28.0) | .742 |
| Completed high school | 157 (31.7) | 57 (39.8) | 100 (28.3) | **.026** |
| Married | 30 (6.0) | 11 (7.7) | 19 (5.4) | .407 |
| Being a parent | 77 (15.5) | 19 (13.3) | 58 (16.4) | .413 |
| *Premorbid functioning, mean (SD)* | | | | |
| Premorbid social functioning^1^ | 0.73 (0.58) | 0.72 (0.57) | 0.73 (0.58) | .975 |
| Premorbid academic functioning^2^ | 0.99 (0.64) | 0.97 (0.62) | 1.01 (0.65) | .527 |
| *Living conditions, n (%)* |  |  |  |  |
| Independent living | 376 (75.8) | 118 (82.5) | 258 (73.1) | .304 |
| Living with parents | 83 (16.7) | 18 (5.6) | 65 (18.4) |  |
| Living in supported housing | 3 (0.6) | 0 (0.0) | 3 (0.8) |  |
| Homelessness | 26 (5.2) | 7 (4.9) | 19 (5.4) |  |
| *Symptom dimension, mean (SD)* | | | | |
| Psychotic dimension^3^ | 3.00 (1.30) | 3.00 (1.38) | 3.00 (1.29) | .952 |
| Negative dimension^4^ | 2.21 (1.16) | 2.00 (1.12) | 2.30 (1.17) | **.010** |
| Disorganized dimension^5^ | 1.06 (0.96) | 0.90 (0.93) | 1.13 (0.97) | **.019** |
| *Global functioning score, mean (SD)* | | | | |
| GAF-F^6^, Function scale | 39.80 (13.10) | 42.88 (13.60) | 38.48 (12.76) | **.001** |
| GAF-S^7^, Symptom scale | 31.30 (9.70) | 32.72 (9.68) | 30.76 (9.65) | **.042** |
| *Antipsychotic treatment (at one year follow-up), n (%)* | | | | |
| Antipsychotic use | 310 (62.5) | 89 (62.2) | 221 (62.6) | .838 |
| *Diagnoses, n (%)* | | | | |
| Alcohol/substance use disorder | 144 (29.0) | 41 (28.6) | 102 (28.9) | .913 |
| Schizophrenia (F20) | 388 (78.2) | 113 (79.0) | 275 (77.9) | .932 |
| Others (F22-25)(F28-F29) | 108 (21.8) | 31 (21.7) | 77 (21.8) |  |
| *Duration of untreated psychosis (DUP in weeks), mean (SD)^8^* | | | | |
| DUP | 124.89 (186.02) | 122.57 (186.03) | 125.31 (186.29) | .884 |

^1^ Premorbid Adjustment Scale (Premorbid social functioning)

^2^ Premorbid Adjustment Scale (Premorbid academic functioning)

^3^ Scale for Assessment for Positive Symptoms (SAPS)

^4^ Scale for Assessment of Negative Symptoms (SANS)

^5^ Disorganized symptoms were assessed using SANS and SAPS

^6^ Global Assessment of Functioning scale

^7^ GAF-S

^8^ Duration of Untreated Psychosis (DUP)

| **eTable 2. 10-year characteristics among participants and non-participants at the 20-year follow-up** | | | | |
| --- | --- | --- | --- | --- |
| ***10-year data*** | All,  n= 291 | 20y-Participants,  n=122 | 20y-non-Participants,  n=169 | P value |
| *Sociodemographics, n (%)* | | | | |
| Sex, Female | 129 (44.3) | 63 (51.6) | 66 (39.1) | **.033** |
| *Symptom dimension, mean (SD)* | | | | |
| Psychotic dimension^1^ | 1.25 (1.45) | 0.98 (1.41) | 1.44 (1.45) | **.008** |
| Negative dimension^2^ | 1.50 (1.11) | 1.22 (0.99) | 2.30 (1.17) | **.021** |
| Disorganized dimension^3^ | 0.54 (0.72) | 0.43 (0.67) | 1.69 (1.16) | **.001** |
| *Global functioning score, mean (SD)* | | | | |
| GAF-F^4^, Function scale | 53.11 (16.22) | 58.25 (15.65) | 49.43 (15.65) | **.001** |
| *Antipsychotic treatment (at the 10-year follow-up), n (%)* | | | | |
| Antipsychotic use | 176 (60.5) | 65 (53.3) | 111 (65.7) | **.033** |
| *Diagnoses at baseline, n (%)* | | | | |
| Alcohol/substance use disorder | 91 (31.3) | 38 (31.1) | 53 (31.4) | .969 |
| Schizophrenia (F20) | 232 (79.7) | 97 (79.5) | 135 (79.9) | .938 |
| Others (F22-25)(F28-F29) | 59 (20.3) | 25 (20.5) | 34 (20.1) |  |
| *Duration of untreated psychosis (DUP in weeks), mean (SD)^5^* | | | | |
| DUP | 116.33 (173.06) | 116.54 (180.33) | 116.18 (167.99) | .889 |
| Remission status and use of antipsychotic medication at the 10 year follow-up, n (%) | | | | |
| Remission-of-psychotic-symptom-off-medication* | 87 (29.9) | 46 (37.7) | 41 (24.3) | **.009** |
| Remission-of-Psychotic-symptom-on-medication* | 85 (29.2) | 39 (32.0) | 46 (27.2) |  |
| Non-remission-of-psychotic-symptom-off-medication* | 28 (9.6) | 11 (9.0) | 17 (10.0) |  |
| Non-remission-of-psychotic-symptom-on-medication* | 91 (31.3) | 26 (21.3) | 65 (38.5) |  |

^1^ Scale for Assessment for Positive Symptoms (SAPS)

^2^ Scale for Assessment of Negative Symptoms (SANS)

^3^ Disorganized symptoms were assessed using SANS and SAPS

^4^ Global Assessment of Functioning scale

^5^ Duration of Untreated Psychosis (DUP)

* Remission-of-psychotic-symptoms-off-medication, Remission-of-psychotic-symptoms-on-medication, Non-remission-of-psychotic-symptom-off-medication, and Non-remission-psychotic-symptoms-on- medication were based on all items on Scale for Assessment for Positive Symptoms (SAPS) and use of antipsychotic medication. To be considered non-psychotic and in remission, the global scores for hallucinations, delusions, bizarre behavior, and thought disorder should be two or less for six consecutive months in accordance with the Remission in Schizophrenia Working Group

**eFigure 1:** Flow-chart of participation


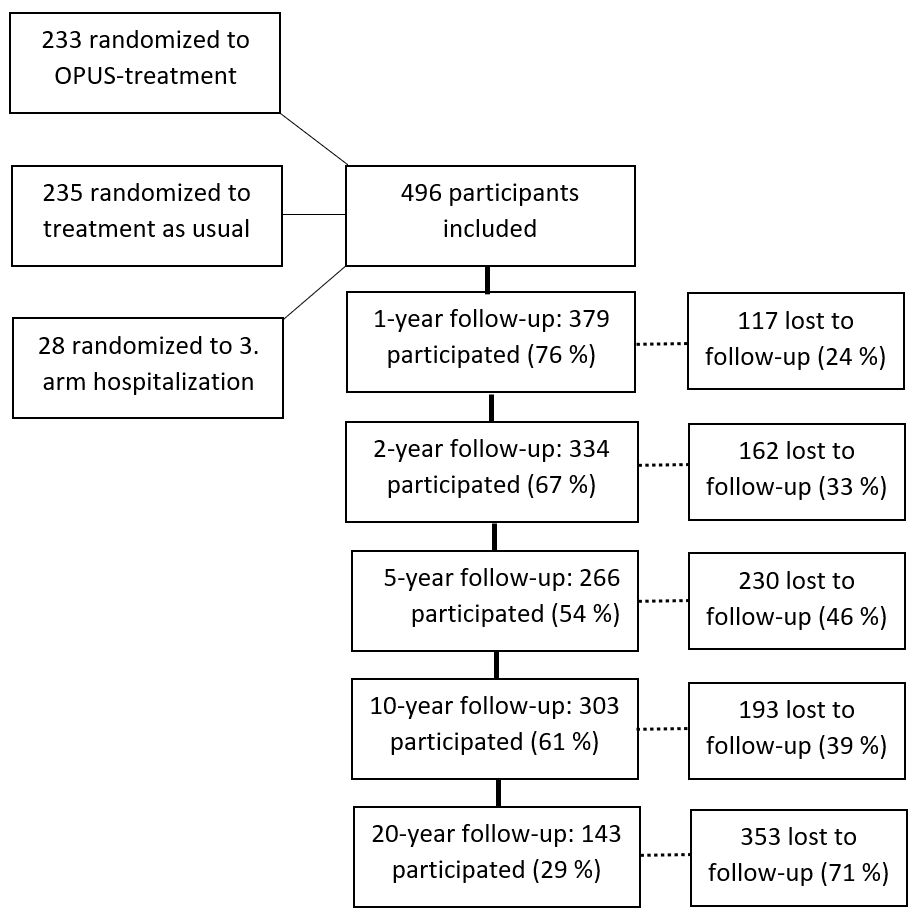


*Number of lost to follow-up refers only to the specific follow-up year and participants could continue to be contacted and contribute to the study longitudinally

**eFigure 2.** Proportional flow between users and no use of antipsychotic medication through a period of 20 years among 496 individuals with a first episode of schizophrenia.


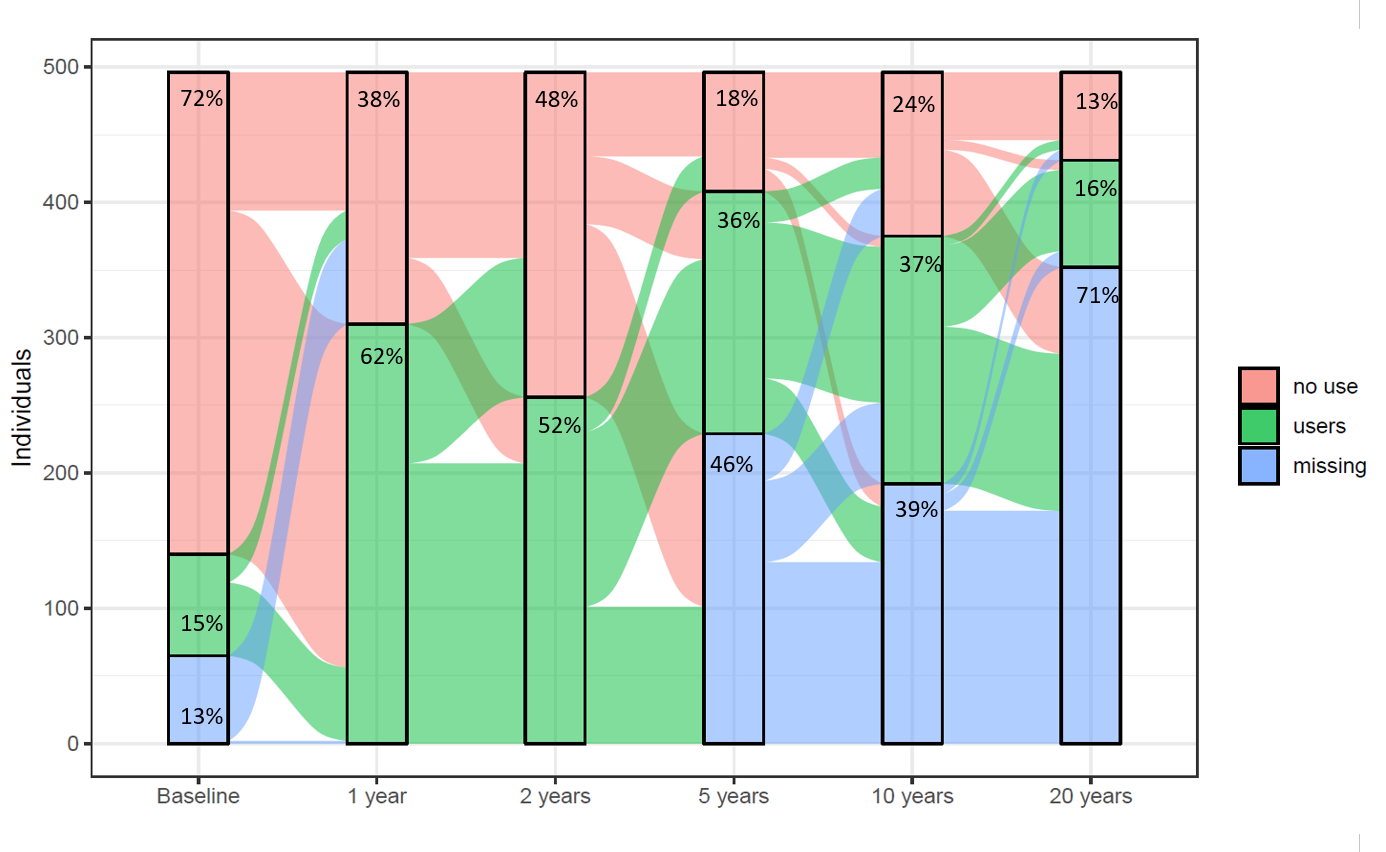


|  | **Baseline** | **1 yr** | **2yr** | **5 yr** | **10 yr** | **20 yr** |
| --- | --- | --- | --- | --- | --- | --- |
| **No use,** n (%) | 356 (71.8) | 186 (37.5) | 240 (48.4) | 88 (17.7) | 121 (24.4) | 65 (13.1) |
| **Users,** n (%) | 75 (15.1) | 310 (62.5) | 256 (51.6) | 179 (36) | 183 (36.9) | 79 (15.9) |
| **Missing,** n (%) | 65 (13.1) | 0 (0) | 0 (0) | 229 (46.2) | (192 (38.7) | 352 (71) |
